# The Skin Antiseptic agents at Vaginal dElivery (SAVE) trial: study protocol for a randomized controlled trial

**DOI:** 10.1186/s13063-023-07101-w

**Published:** 2023-02-21

**Authors:** Young Mi Jung, Seung Mi Lee, So Yeon Kim, Jin Hoon Chung, Hye-Sung Won, Kyung A Lee, Mi Hye Park, Geum Joon Cho, Min-Jeong Oh, Eun Saem Choi, Ki Hoon Ahn, Soon-Cheol Hong, Ji-Hee Sung, Cheong-Rae Roh, Sun Min Kim, Byoung Jae Kim, Hyeon Ji Kim, Kyung Joon Oh, Subeen Hong, In Yang Park, Joong Shin Park

**Affiliations:** 1 Department of Obstetrics and Gynecology, Seoul National University College of Medicine, 101 Daehak-ro, Jongno-gu, Seoul, 03080 South Korea; 2 Department of Obstetrics and Gynecology, University of Ulsan College of Medicine, Asan Medical Center, Seoul, South Korea; 3 Department of Obstetrics and Gynecology, Ewha Womans University Seoul Hospital, College of Medicine, Ewha Womans University, Seoul, South Korea; 4 Department of Obstetrics and Gynecology, Guro Hospital, College of Medicine, Korea University, Seoul, South Korea; 5 Department of Obstetrics and Gynecology, Korea University College of Medicine, Seoul, South Korea; 6 Department of Obstetrics and Gynecology, Korea University Anam Hospital, Seoul, South Korea; 7 Department of Obstetrics and Gynecology, Samsung Medical Center, Sungkyunkwan University School of Medicine, Seoul, South Korea; 8 Department of Obstetrics and Gynecology, Seoul Metropolitan Government, Seoul National University Boramae Medical Center, Seoul, South Korea; 9 Department of Obstetrics and Gynecology, Seoul National University Bundang Hospital, Seongnam-si, Gyeonggi-do South Korea; 10 Department of Obstetrics and Gynecology, Seoul St. Mary’s Hospital, College of Medicine, The Catholic University of Korea, Seoul, South Korea

**Keywords:** Antiseptic, Perineal infection, Vaginal delivery, Randomized controlled trial

## Abstract

**Background:**

Cleansing of the vulva and perineum is recommended during preparation for vaginal delivery, and special attention is paid to cleansing before episiotomy because episiotomy is known to increase the risk of perineal wound infection and/or dehiscence. However, the optimal method of perineal cleansing has not been established, including the choice of antiseptic agent. To address this issue, we designed a randomized controlled trial to examine whether skin preparation with chlorhexidine-alcohol is superior to povidone-iodine for the prevention of perineal wound infection after vaginal delivery.

**Methods:**

In this multicenter randomized controlled trial, term pregnant women who plan to deliver vaginally after episiotomy will be enrolled. The participants will be randomly assigned to use antiseptic agents for perineal cleansing (povidone-iodine or chlorhexidine-alcohol). The primary outcome is superficial or deep perineal wound infection within 30 days after vaginal delivery. The secondary outcomes are the length of hospital stay, physician office visits, or hospital readmission for infection-related complications, endometritis, skin irritations, and allergic reactions.

**Discussion:**

This study will be the first randomized controlled trial aiming to determine the optimal antiseptic agent for the prevention of perineal wound infections after vaginal delivery.

**Trial registration:**

ClinicalTrials.gov NCT05122169. First submitted date on 8 November 2021. First posted date on 16 November 2021

## Administrative information

Note: the numbers in curly brackets in this protocol refer to SPIRIT checklist item numbers. The order of the items has been modified to group similar items (see http://www.equator-network.org/reporting-guidelines/spirit-2013-statementdefning-standard-protocol-items-for-clinical-trials/).

**Table:** 

Title {1}	The Skin Antiseptic agents at Vaginal dElivery (SAVE) trial
Trial registration {2a and 2b}	ID: NCT05122169, ClinicalTrials.gov, first submitted date: November 8, 2021, first posted date: November 16, 2021
Protocol version {3}	Version 1.0.9, 5 December 2022
Funding {4}	This research was supported by a grant of Patient-Centered Clinical Research Coordinating Center (PACEN) funded by the Ministry of Health & Welfare, Republic of Korea (grant number : HC21C0090).
Author details {5a}	^1^Department of Obstetrics and Gynecology, Seoul National University College of Medicine, Seoul, Korea; ^2^Department of Obstetrics and Gynecology, University of Ulsan College of Medicine, Asan Medical Center, Seoul, Korea; ^3^Department of Obstetrics and Gynecology, Ewha Womans University Seoul Hospital, College of Medicine, Ewha Womans University, Seoul, Korea; ^4^Department of Obstetrics and Gynecology, Guro Hospital, College of Medicine, Korea University, Seoul, Korea; ^5^Department of Obstetrics and Gynecology, Korea University College of Medicine, Seoul, Korea; ^6^Department of Obstetrics and Gynecology, Korea University Anam Hospital, Seoul, Korea; ^7^Department of Obstetrics and Gynecology, Samsung Medical Center, Sungkyunkwan University School of Medicine, Seoul, Korea; ^8^Department of Obstetrics and Gynecology, Seoul Metropolitan Government, Seoul National University Boramae Medical Center, Seoul, Korea; ^9^Department of Obstetrics and Gynecology, Seoul National University Bundang Hospital, Seongnam-si, Gyeonggi-do, Korea; ^10^Department of Obstetrics and Gynecology, Seoul St. Mary’s Hospital, College of Medicine, The Catholic University of Korea, Seoul, Korea
Name and contact information for the trial sponsor {5b}	Patient-Centered Clinical Research Coordinating Center (PACEN) funded by the Ministry of Health & Welfare, Republic of Korea Seung Tae Kim, e-mail: stkim@neca.re.kr
Role of sponsor {5c}	The funding body had no role in the design of the protocol and will not have any role in the conduct of the study including collection, analysis, and interpretation of data, or the writing of the manuscript or the decision to publish.

## Background and rationale {6a}

Perineum repair, with or without an episiotomy, is one of the most common surgical procedures for women with damage to the labia, vagina, or perineum during vaginal delivery. Perineal trauma and its repair are strongly associated with postnatal morbidity, including bleeding, infection, pain, urinary and fecal incontinence, and sexual dysfunction [1, 2]. Some studies have shown that episiotomy inevitably leads to an incisional infection [3, 4].

To avoid severe tears and facilitate birth, surgical incision of the perineum (i.e., episiotomy) is often needed before the delivery of the fetal head [5]. Episiotomy rates vary considerably between countries. In Taiwan and China, it has been reported that episiotomy is performed during most vaginal deliveries [6, 7]. However, routine episiotomy is no longer recommended; instead, episiotomy is mainly applied for selective indications such as shoulder dystocia, breech delivery, fetal macrosomia, operative vaginal delivery, persistent occiput posterior position, and a markedly short perineal length [5]. Episiotomy leads to incisional infections due to the specific anatomical position of the incision, which confers susceptibility to vaginal, intestinal, and urethral microbial flora infection [8].

Surgical site infections, which are the second most common type of healthcare-associated infections, are preventable problem that incurs high medical costs. Episiotomy site infection complicates 2.5~9.5% of vaginal deliveries [9, 10]. The skin is a major source of pathogens that cause surgical site infections. The main pathogens involved in perineal infections are *Pseudomonas aeruginosa*, *Escherichia coli*, *Staphylococcus aureus*, and *Staphylococcus epidermidis* [9].

Perineal cleansing before vaginal delivery is one of the steps in preparation for delivery. Perineal antiseptic skin preparation attempts to achieve a sterile field by decreasing the concentration of bacteria colonizing the skin at the episiotomy site. Therefore, optimizing skin antisepsis has the potential to reduce the rate of perineal site infections. However, there is a paucity of information supporting the choice of specific antiseptic agents for vaginal delivery.

### Objectives {7}

The aim of this study is to examine whether skin preparation with chlorhexidine-alcohol is superior to povidone-iodine for the prevention of perineal wound infections after vaginal delivery.

### Trial design {8}

**Fig. 1:**
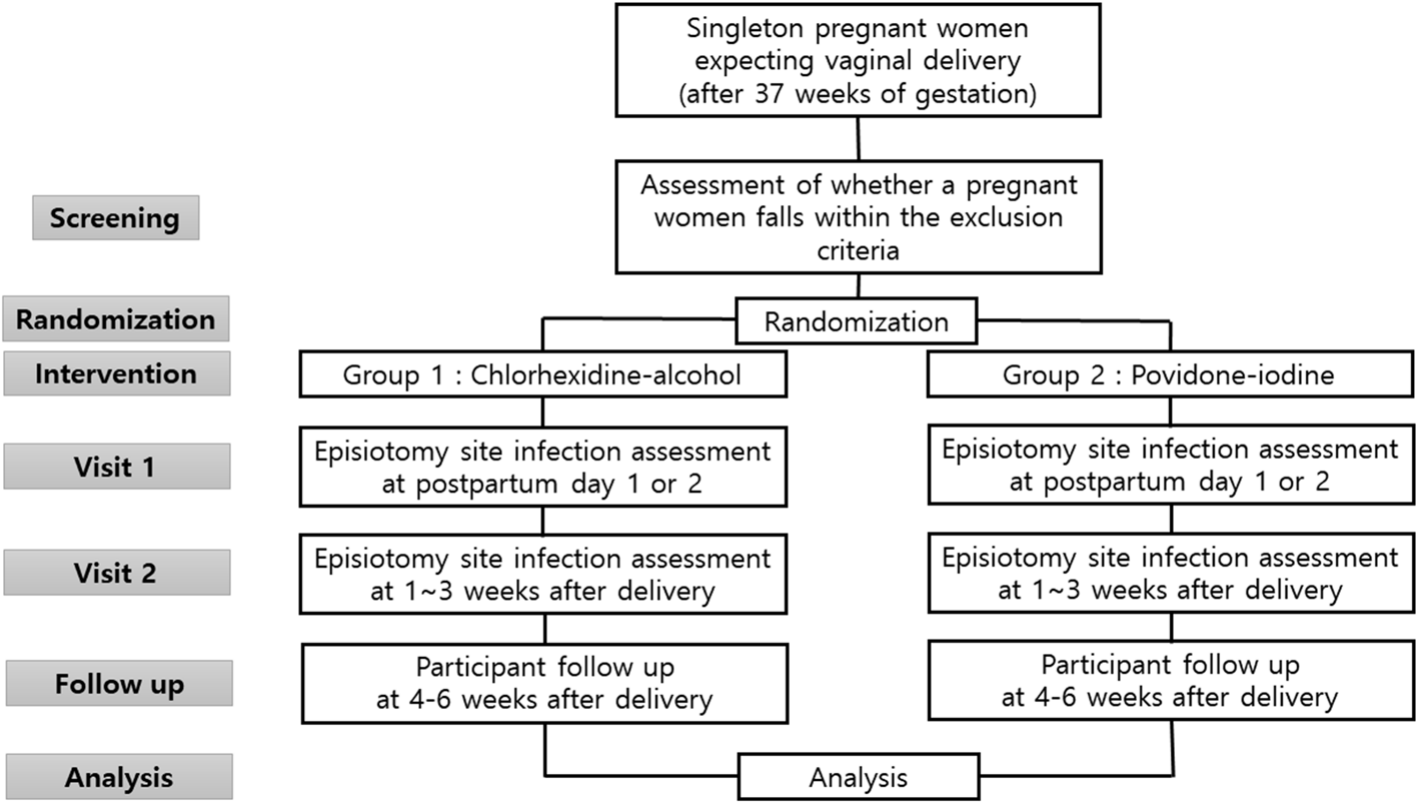
Study flowchart

This multicenter, randomized, single-blind placebo-controlled trial (SAVE trial) will enroll women undergoing vaginal delivery. Figure 1 shows a schematic diagram of the study design. After providing written informed consent, women will be randomly assigned to skin antisepsis with chlorhexidine-alcohol or povidone-iodine. Except for the preparation of skin before vaginal delivery, the participants will be treated according to obstetric guidelines at the discretion of the attending physician.

## Methods: participants, interventions, and outcomes

### Study setting {9}

This study will be conducted at the obstetric departments of nine hospitals in South Korea: Asan Medical Center, Ewha Womans University Seoul Hospital, Korea University Guro Hospital, Korea University Anam Hospital, Samsung Seoul Hospital, Seoul National University Hospital, Seoul National University Boramae Medical Center, Seoul National University Bundang Hospital, and Seoul St. Mary’s Hospital. These are all tertiary referral hospitals.

### Eligible criteria {10}

Korean women with term singleton pregnancies who plan to deliver vaginally following episiotomy will be enrolled. The exclusion criteria will be as follows: (1) allergy to chlorhexidine, alcohol, iodine, or shellfish and (2) evidence of infection of the perineum (Table 1). After screening for eligibility, information regarding the study will be provided, and written informed consent will be obtained. The dropout criteria will include the patient’s withdrawal of content, the occurrence of a severe adverse reaction, or a clinical situation that does not permit the continuation of the trial protocol at the discretion of the investigators.

**Table 1:** Inclusion and exclusion criteria

Inclusion criteria	Exclusion criteria
Singleton pregnancy Women expecting vaginal delivery after 37 weeks	Allergy to chlorhexidine, alcohol, iodine, or shellfish Women who have evidence of infection of the perineum

### Who will take informed consent? {26a}

Consent for the trial will be obtained in writing using a paper consent form. All pregnant women will be given the opportunity to ask questions prior to completing their consent form. Consent will be obtained by a member of the research team. Prior to randomization, each pregnant women will be checked for eligibility and documented on the checklist.

### Additional consent provisions for collection and use of participant data and biological specimens {26b}

This trial does not involve collection of biological specimens.

### Interventions

#### Explanation for the choice of comparators {6b}

The chlorhexidine-alcohol and povidone-iodine have been approved by Food and Drug as antiseptics for skin. Chlorhexidine and iodine-based preparations have both been shown to decrease bacterial counts and are widely used.

#### Intervention description {11a}

The chlorhexidine-alcohol antiseptic will be 2% chlorhexidine gluconate with 72% alcohol, and the povidone-iodine antiseptic will be 10% povidone-iodine. The eligible participants for the inclusion and exclusion criteria will be randomized into two groups: group 1, chlorhexidine-alcohol group, and group 2, povidone-iodine group.

#### Criteria for discontinuing or modifying allocated interventions {11b}

Participants of the study can withdraw their consent to take part at any time. The principal investigator may exclude patients from the study, if patients’ safety is at risk. In order to generate a meaningful database, excluded patients can be replaced by the recruitment of new patients.

#### Strategies to improve adherence to interventions {11c}

Not applicable as the intervention is performed only once during delivery. Adherence to the follow-up visit schedule is promoted by facilitating the study visit within 3 weeks of after delivery and telephone interview at 4 weeks after delivery.

#### Relevant concomitant care permitted or prohibited during the trial {11d}

Preexisting perineal infection before delivery is excluded, where additional antibiotics treatment after delivery will be documented.

### Post-trial care {30}

There are no provisions for post-trial care.

### Study outcomes {12}

Data on obstetric and neonatal infection outcomes will be gathered. The primary outcome is the proportion of perineal infections (superficial or deep infections within 30 days of vaginal delivery). Perineal infections will be diagnosed by blinded physicians and verified by medical record review in accordance with the CDC Nosocomial Infections Surveillance System definitions (Table 2). The secondary outcomes are the length of hospital stay, number of office visits, and readmissions for infection-related complications, endometritis, positive culture from wound culture, skin irritation, and allergic reactions (Table 3).

**Table 2:** Definition of surgical site infection

**Superficial incisional surgical site infection**	
*An infection that occurs within 30 days after the operation and involves only skin or subcutaneous tissue at the incision and at least one of the following:* 1. Purulent drainage, with or without laboratory confirmation, from the superficial incision. 2. Organisms isolated from an aseptically obtained culture of fluid or tissue from the superficial incision. 3. At least one of the following signs or symptoms of infection: pain or tenderness, localized swelling, redness, or heat, and the superficial incision is deliberately opened by the surgeon, unless the incision is culture-negative. 4. Diagnosis of superficial incisional surgical site infection by the surgeon or attending physician.	
**Deep incisional surgical site infection**	
*An infection that occurs within 30 days after the operation and involves deep soft tissue (e.g., fascial and muscle layers) around the incision and at least one of the following:* 1. Purulent drainage from the deep incision but not from the organ/space component of the surgical site. 2. A deep incision spontaneously dehisces or is deliberately opened by the surgeon when the patient has at least the following signs or symptoms: fever (>38°C), localized pain, or tenderness, unless the site is culture-negative. 3. An abscess or other evidence of infection involving the deep incision is found on direct examination, during reoperation, or by a histopathological or radiological examination. 4. Diagnosis of superficial incisional surgical site infection by the surgeon or attending physician.

**Table 3:** Primary and secondary outcomes

Primary outcome (superficial or deep infection within 30 days of vaginal delivery)	Secondary outcome
Proportion of perineal infections	Length of hospital stay Number of office visits and readmissions for infection-related complications
	Endometritis Positive culture from the wound culture Skin irritation Skin allergic reaction

### Participant timeline {13}

Participants are recruited into the trial for a total of 4–6 weeks following randomization. The summary of trial procedures is detailed in Fig. 1.

### Sample size {14}

The sample size was calculated to determine how many participants would be needed to detect a risk reduction from 6% by chlorhexidine-alcohol to 4% by povidone-iodine. We estimated the perineal infection rate as 6% for povidone-iodine, according to our retrospective database. To have 80% power, a type 1 error of 0.05, and a ratio of 1:1 between chlorhexidine-alcohol and 4% by povidone-iodine, a total of 3726 subjects will need to be randomized. To accommodate a 10% dropout rate, 4140 subjects will be enrolled (2070 chlorhexidine-alcohol, 2070 povidone-iodine). The sample size was calculated (PASS 15 (NCSS Statistical Software, USA)) based on the primary endpoint of the study.

### Recruitment {15}

The recruitment period is expected to be approximately 42 months (first patient in, to last patient out 43 months). In the study sites, an average of about 180 vaginal deliveries are performed per month. Considering the dropout rate, it was calculated that about 15 patients per institution would be enrolled in 1 month. To cover any unforeseen recruitment difficulties, for example due to the COVID-19 pandemic, 6 extra months of recruitment time were added.

## Assignment of interventions: allocation

### Sequence generation {16a}

Participants will be assigned to intervention groups at a 1:1 ratio (stratified by the institution) to chlorhexidine-alcohol (group 1) or povidone-iodine (group 2) using an Internet-based randomization system developed and maintained by the Medical Research Collaborating Center (MRCC) of Seoul National University Hospital. To reduce the predictability of random sequence, stratified block randomization method will be used.

### Concealment mechanism {16b}

Allocation will be concealed to all trial staff using an automated web system operated by the MRCC.

### Implementation {16c}

An authorized unblinded researchers will log into the secure randomization web system and randomize the participant following completion of informed consent and confirmation of eligibility. Unblinded researchers will not participate in the subsequent process of data management and data analysis. The unblinded researchers will randomly assign the patients and prepare the skin with the assigned antiseptic agents.

## Assignment of interventions: blinding

### Who will be blinded {17a}

Blinding of the participant and site research team is not possible due to the color difference of the antiseptic agents; a double-blinded study is not feasible in the current study. Unblinded researchers will not participate in the subsequent process of data management and data analysis. Researchers assessing outcomes will be blinded to the participant’s allocation.

### Procedure for unblinding if needed {17b}

The design is open label with only outcome assessors being blinded so unblinding will not occur.

## Data collection and management

### Plans for assessment and collection of outcomes {18a}

Most of the outcomes are collected in the 4 weeks after delivery with physical exam of the perineum, hospital records, and questionnaires. In cases where participants do not come to a follow-up visit, adherence data will be collected rescheduled outpatient clinic.

### Plans for promoting participant retention and complete follow-up {18b}

Strategies to minimize loss to follow-up will include using text message and phone reminders made to participants.

### Data management {19}

All baseline data will be collected by the site staff and entered onto iCREAT database. This system is developed and maintained by the Korea National Institute of Health. Access to the database will be restricted and secure. Sites will be provided with workbooks to assist other participant sites with data collection. Missing or spurious data will be queried in a timely manner throughout the trial period.

### Confidentiality {27}

Participant’s medical information obtained as a result of this trial is considered confidential and disclosure to third parties is prohibited with the exceptions noted in this protocol.

### Plans for collection, laboratory evaluation, and storage of biological specimens for genetic or molecular analysis in this trial/future use {33}

Not applicable since no biological specimen will be taken.

## Statistical methods

### Analysis {20a}

The efficacy of both antiseptic agents will be assessed by comparing the primary and secondary outcomes between the groups. Efficacy analysis will be conducted based on the intention-to-treat (ITT) and per-protocol (PP) principles, with a primary consideration for ITT results. A *p*-value of less than 0.05 will be considered to indicate statistical significance, and incidence rate and 95% confidence intervals will be reported. It will be compared with logistic regression analysis adjusting for the institution. A safety analysis will be performed based on the safety group, which will include participants who have been administered with at least one dose of the antiseptic agents. All analyses will be performed using SAS statistics software version 9.2.

### Interim analyses {21b}

There will be no planned interim effectiveness analyses. However, an internal pilot phase will be performed to check the feasibility of recruitment.

### Methods for additional analyses {20b}

The trial is powered to detect overall differences between groups. Therefore, any sub-group analyses will be regarded as exploratory.

### Methods in analysis to handle protocol non-adherence and any statistical methods to handle missing data {20c}

We plan to conduct a per-protocol group analysis as a sensitivity analysis. For the primary outcome analysis, missing values will be imputed using the multiple imputation method in the intention-to-treat analysis.

### Plans to give access to the full protocol, participant-level data, and statistical code {31c}

The full protocol and statistical code can be requested from the authors. Individual participant-level data can be shared after anonymization, with investigators who are approved by an independent review committee.

## Oversight and monitoring

### Composition of the coordinating center and trial steering committee {5d}

Initially, on-site monitoring of the eligibility for recruited participants will take place at least annually at the lead site (Seoul National University Hospital) but this may be increased/reduced as deemed necessary. Other than this, on-site monitoring will not be conducted routinely throughout the trial. Central monitoring of all trial data (across recruiting centers and participant-reported data) will be undertaken and used to assess whether sites have met any of the monitoring triggers detailed in the trial monitoring plan. The Medical Research Cooperation Center (MRCC) will review ongoing trial data about 1–2 times per year.

### Composition of the data monitoring committee, its role, and reporting structure {21a}

The data will be monitored at the MRCC of Seoul National University Hospital, an institution independent of the researchers.

### Adverse event reporting and harms {22}

Solicited and spontaneously reported adverse events will be collected through electronic-case report form and analyzed and presented. To provide adverse event data associated with the use of chlorhexidine-alcohol or povidone-iodine, only specified adverse reactions experienced during treatment will be collected. Serious adverse events (SAEs) are not expected in this trial; however, we will collect details of SAEs experienced after delivery. If there is a SAE, it will be reported to the IRB within 15 days*.*

### Frequency and plans for auditing trial conduct {23}

A review of trial conduct will be taken by the monitoring manager according to the monitoring plan. Auditing by an external institution may be conducted if necessary.

### Plans for communicating important protocol modification {25}

Any amendments made to the trial protocol will undergo review and approval by the sponsor, MRCC, Research Ethics Committee prior to modification. Updated versions of the protocol will be shared with recruiting centers via email and uploaded to the trial website.

### Dissemination plan {31a}

Trial results are submitted for publication in peer-reviewed journals and published as reports by the Patient-Centered Clinical Research Coordinating Center.

## Discussion

The purpose of this trial is to determine which antiseptic is superior for preventing perineal infection after vaginal delivery. In clinical practice, both chlorhexidine-alcohol and povidone-iodine are used to reduce surgical site infection. Several previous studies have compared the effectiveness of antiseptic agents in obstetric and gynecological surgery, including cesarean sections [11, 12]. However, no prospective randomized controlled trial has yet investigated the relative effectiveness of chlorhexidine and iodine skin preparations for reducing skin contamination and perineal site infections.

Episiotomy site infection, which complicates 2.5~9.5% of vaginal deliveries [9, 10], is associated with significant morbidity, including additional hospital costs [13]. The use of antiseptic agents prior to vaginal delivery is an important intervention for reducing the risk of perineal infection by decreasing the concentration of skin-colonized bacteria. The Food and Drug Administration has approved several antiseptic agents, including iodine, chlorhexidine, and alcohol, for skin antisepsis. Iodine-based preparations and chlorhexidine have been shown to decrease bacterial concentrations and are widely used.

Iodine acts by oxidizing sulfhydryl groups and destroying microbial protein structures. The potential disadvantages of iodine are skin irritation and a relatively long drying time for optimal action. In contrast, chlorhexidine does not require a wait time between application and surgical incision. However, it is more expensive than iodine and may be associated with an increased risk of allergic reactions. Chlorhexidine acts by destroying bacterial cell membranes and precipitating the cell contents. Alcohol is believed to act by damaging microbial cell membranes and denaturing proteins. It has the advantage of being broad-spectrum and fast-acting, but lacks persistent activity [14]. Due to this problem, it is mainly used in combination with other antiseptic agents. The use of both antiseptic agents varies at different hospitals, and there is a paucity of information to recommend the choice of antiseptic agents for vaginal delivery. In this regard, research on this issue is anticipated to play a crucial role in clinical practice.

The results of this study are expected to have a substantial impact on several issues. First, this study may have a large effect on patient outcomes. Identifying which antiseptic agent is more effective will lead to changes in practice. The results of this study will also provide evidence for clinical guidelines on perineal preparation for vaginal delivery.

Second, this study design has important methodological strengths. This is a single-blind, randomized controlled study, which provides a high level of evidence. In addition, the objectivity of the evaluation variable is expected to produce objective results.

Third, the results of this study will have a significant impact on improving national health and related policies. Identifying which antiseptic agent is more effective will reduce perineal site infections and the subsequent burden due to increased hospital costs.

## Trial status

Enrollment is ongoing. Recruitment started in March 2022 and is expected to conclude in December 2025. The target enrollment for the study is 4140 participants. Protocol version: ver 1.0.9

## Data Availability

Any data required to support the protocol can be supplied on request.

## Abbreviations

CDC: Centers for Disease Control and Prevention
ITT: Intention-to-treat
MRCC: Medical Research Cooperation Center
PP: Per-protocol
SAEs: Serious adverse events
